# What “Tears” Remind Us of: An Investigation of Embodied Cognition and Schizotypal Personality Trait Using Pencil and Teardrop Glasses

**DOI:** 10.3389/fpsyg.2019.02826

**Published:** 2020-01-10

**Authors:** Yu Liang, Kazuma Shimokawa, Shigeo Yoshida, Eriko Sugimori

**Affiliations:** ^1^Graduate School of Human Sciences, Waseda University, Tokyo, Japan; ^2^Cyber Interface Laboratory, The University of Tokyo, Tokyo, Japan

**Keywords:** embodied cognition, facial feedback, congruence hypothesis, schizotypal personality trait, schizotypy, sense of agency/ownership, teardrop glasses

## Abstract

Facial expressions influence our experience and perception of emotions—they not only tell other people what we are feeling but also might tell us what to feel *via* sensory feedback. We conducted three experiments to investigate the interaction between facial feedback phenomena and different environmental stimuli, by asking participants to remember emotional autobiographical memories. Moreover, we examined how people with schizotypal traits would be affected by their experience of emotional facial simulations. We found that using a directed approach (gripping a pencil with teeth/lips) while remembering a specific autobiographical memory could successfully evoke participants’ positive (e.g., happy and excited)/negative (e.g., angry and sad) emotions (i.e., Experiment 1). When using indirective environmental stimuli (e.g., teardrop glasses), the results of our experiments (i.e., Experiments 2 and 3) investigating facial feedback and the effect of teardrop glasses showed that participants who scored low in schizotypy reported little effect from wearing teardrop glasses, while those with high schizotypy reported a much greater effect in both between- and within-subject conditions. The results are discussed from the perspective of sense of ownership, which people with schizophrenia are believed to have deficits in.

## Introduction

Understanding the relationship between physiological changes and emotion has enticed scientists for a very long time. In writing his theory of evolution, Darwin and Prodger (1998) proposed the foremost statement about the physical body and emotion, stating that facial expressions developed because they were serviceable habits or gestures that solved a problem in our evolutionary past (Darwin and Prodger, 1998; Hess and Thibault, 2009; Niedenthal and Ric, 2017). William James was quite clear in his emotion theory that expressive behavior like crying and facial expressions like furrowed brows contributed to the experience of various emotions (James, 1884; Laird and Lacasse, 2014). James proposed that emotions take place when we perceive particular facets of our environment that generate a collection of physical changes, and that “our feeling of the same changes as they occur is the emotion” (Prinz, 2004). At the same time, Lange proposed that all emotions are developed from, and can be reduced to, physiological reactions to a given stimulus. Taking their work together – referred to as the James-Lange theory (Cannon, 1927) – emotions can be defined as feelings resulting from the physiological changes that arise in response to a stimulus.

Many studies have investigated the relationship between emotions and subsequent physical changes. In particular, many scientists have come to believe that our emotions are affected by our facial expressions. Emotion theorists (Tomkins, 1962; Izard, 2013) later developed the idea that facial behavior can activate or regulate the expression of emotion. The facial feedback hypothesis states that skeletal muscle feedback from facial expressions causes the regulation of emotional experiences and behavior. This is an important part of several contemporary theories of emotion (Buck, 1980).

In an early study of this phenomenon, participants were told that the purpose was to measure the electromyographic changes of facial muscles that accompanied perceptual tasks (Laird and Crosby, 1974). To this end, electrodes were attached to their faces, and they were asked to contract or relax the muscles while wearing different electrodes, which led them to form different facial expressions. In interviews about their understanding of the experiment, they tended to report feelings consistent with their expressions. In a study where participants were asked to either conceal or exaggerate facial displays associated with the anticipation and reception of painful shocks in intensity, results showed that the suppression of expressive responses decreased the magnitude of phasic skin conductance changes and subjective reports of painfulness when contrasted with free expression or exaggeration of pain-related expressive responses (Lanzetta et al., 1976). Primary research on the facial feedback hypothesis focused on the enhancing or suppressing effect of facial changes and emotional feedback. For the most part, the purpose of these studies was obvious until Strack et al. (1988) tested the theory with a solely physical facial change, using only certain facial muscles. Strack found that physical changes in facial expression (e.g., gripping a pencil with one’s teeth) could unconsciously induce emotional arousal (e.g., feelings of happiness). Additionally, Zajonc et al. (1989) conducted research that found that participants’ reactions that resulted from facial actions (namely, phonetic utterance) resembling but unrelated to emotional efference differed in hedonic qualities and produced correlated changes in forehead temperatures. Similarly, Soussignan (2002) examined the muscles involved in facial expressions and obtained results congruent with the facial feedback hypothesis; namely, facial feedback occurred when the facial structure formed a valid analog of a basic emotional expression.

Another experiment offering support for the facial feedback mechanism was provided using the toxin botulinum. The results of Lewis and Bowler (2009) suggested that botulinum could be used as a treatment for depression. Furthermore, Hennenlotter et al. (2008) studied facial feedback effects on limbic brain responses during the intentional imitation of facial expressions, applying botulinum toxin (BTX)-induced denervation of the muscles involved in frowning, combined with functional magnetic resonance imaging that was used as a reversible lesion model, to minimize afferent muscular and cutaneous input. Results showed that, during the imitation of angry facial expressions, feedback reduced by BTX treatment attenuated the activation of the left amygdala and its functional coupling with brain stem regions that were indicated to be involved in autonomic manifestations of emotional states. A later study on how facial feedback relates to emotion comprehension was performed by Neal and Chartrand (2011) and reached a similar conclusion.

On the contrary, opinions that disagreed with the facial feedback hypothesis, as well as doubts about it, arose in 2016 when a series of replications of the original 1988 experiment conducted in 17 labs did not find support for the hypothesis (Wagenmakers et al., 2016). While in the recent study, facial feedback theory was fairly supported when asking participants gripping pencil in their mouths and facial feedback effects were particularly greater in the presence of certain stimulus types (e.g., imagined scenarios, which were also adopted in the designing of the procedure of the present research) than others (e.g., pictures) (Strack, 2016). Marmolejo-Ramos and Dunn (2013) conducted a study that closely investigated the stimulus types that would likely drive facial feedback, finding that participants’ sensorimotor systems were active when they were asked to judge emotional sentences. Even though that dual activation is not necessary for perceptual and motor systems, it was assumed that the perceptual system mainly drives this cognitive processing. Similarly, in another study, it was found that facial feedback has a stronger effect on emotional experience than do emotionally evocative stimuli (e.g., cartoons) and has a stronger effect when participants were also presented with emotionally evocative stimuli such as emotional sentences (Coles et al., 2019). Additionally, Noah et al. (2018) replicated the facial feedback experiment in two conditions: one with a video camera and one without it. The results revealed a significant facial feedback effect in the absence of a camera, which was eliminated in the camera’s presence. Therefore, to achieve our aim of identifying the relationship between facial feedback phenomena and external stimuli, we adopted the pencil and teardrop glasses as stimuli. Participants were prompted to remember an emotional autobiographical scene to evoke emotional feedback, with no camera present in the environment.

We arranged the first experiment to start discovering the interaction between facial feedback phenomena and stimuli in the environment, on the assumption that there is a connection, determine how they interact with each other. In the first experiment, participants were asked to remember an impressive event while we had them simulate a positive/negative facial expression (i.e., holding a pen between the teeth induces a smile; holding it between the lips induces a pout) in order to evoke a consistent emotion (e.g., a happy feeling) with the external environmental stimuli (e.g., holding a pencil to forming smile facial expression).

Interestingly, researchers studying facial feedback found that the effects of positive and negative facial expressions might differ. Alam et al. (2008) examined the injection of botulinum toxin (BTX) in the upper face and its relation to positive and negative emotional states. They believed the injection of BTX might induce positive feelings as it reduces the ability to generate negative facial expressions, and postulated that the injection of BTX reduced negative facial expressions more than it reduced positive expressions. Another study showed that dysphoric individuals reacted less to positive stimuli, while they showed more responsiveness to negative stimuli such as frowning facial expressions (Sloan et al., 2002). The controversy around facial feedback theory and personality traits in the literature inspired us to launch further investigation, and we wonder if personality traits are the key to various reactions to facial feedback.

We postulated that the schizotypy personality trait would offer a reasonable correlation to investigate, as individuals with high schizotypy are sensitive to social emotional feedback, such as criticism, considered to be negative, and praise, considered positive (Premkumar et al., 2019). Moreover, schizotypy is generally considered to be related to a sense of ownership/agency. Early in 1966, a study called the false heartbeat study was conducted by Valins (1966), where participants were shown a series of seminude female photos; those who heard a false heart rate tended to rate the female in the photo as significantly more attractive. Ehrsson et al. (2004) performed studies on the “rubber hand illusion,” which is regarded as a measure of sense of ownership. Subjects with normal brain function were placed with their left hand out of sight. In front of them, a life-like rubber left hand had been placed. Both the hidden left hand and the visible rubber hand were then caressed with a paintbrush. When the two hands were stroked in the same direction simultaneously, subjects began to feel that the rubber hand was theirs. However, if the real and rubber hands were caressed in different directions or non-synchronously, this did not occur.

Therefore, in the second experiment, participants were asked to remember an impressive event while wearing not a pencil, but a tool with indirective emotional reaction – “tears.” We selected teardrop glasses (i.e., water drops coming out of the glasses to induce negative emotion; no water drops coming out of the glasses for a control condition) to observe the relationship between the emotion evoked by the teardrop glasses and participants’ retrieved autobiographical memory. The teardrop glasses, first introduced by Yoshida (2015) from The University of Tokyo, are shaped like a pair of glasses dispensing a tear-like liquid (i.e., heavier than water) near the lacrimal gland when worn on the face. Yoshida examined emotional feedback while wearing these teardrop glasses and found that participants tended to evaluate a neutral scene as sad when wearing them. Even though the tear-like liquid was issued from the glasses – a physical object in the environment – and not from the user’s physical body, it was perceived as a stimulus that aroused a related emotion. More importantly, participants with schizotypal traits were of particular focus here.

While everyone probably experiences a sense of agency at one time or another, as we mentioned before, patients with schizophrenia experience this illusion more than others do. Mirucka (2016) conducted a series of experiments to investigate how often patients with schizophrenia experienced the rubber hand illusion and found that they experience disruptions in the sense of body ownership much more intensively compared to healthy controls. They thought that potential reasons for this result were two characteristic symptoms of schizophrenia: disturbed perception of authorship and feeling of limited control over one’s own body.

The concept of schizotypy was developed by renowned psychologists including Eysenck (1992) and Claridge et al. (1996) who wanted to understand unusual thoughts and behaviors within the framework of personality theory. Schizotypy refers to a continuum of personality characteristics and experiences that range from normal dissociative, imaginative states to extreme states related to schizophrenia. Moreover, schizotypy is also considered to be related to the sense of ownership/agency frequently shown in many psychological studies (Asai et al., 2011; Kallai et al., 2015).

As patients with full-blown schizophrenia experience illusions related to the sense of ownership/agency more than others do, individuals high in schizotypy also experience more illusions related to the sense of ownership/agency than people low in schizotypy. Therefore, wearing the teardrop glasses might make people high in schizotypy feel that the “tears” being dispensed are their own or that they are crying. In the present experiments, we adopted these teardrop glasses, and particularly the tear-like liquid they issue, as stimuli for inducing negative emotion. If negative emotion was indeed facilitated, it would support the congruence hypothesis.

Finally, in Experiment 3, we compared the variety and content of the retrieved memories among participants in the simulated smile and crying conditions, again with a particular focus on participants with schizotypal personality traits.

To summarize, in the present study, we conducted three experiments to determine the interaction between facial feedback phenomena and a directive environmental stimulus (in Experiment 1) and indirective environmental stimuli (in Experiments 2 and 3) by prompting the recall of a specific emotional autobiographical memory. We supposed there to be a connection, determined by how they interact with each other. We further investigated individual differences (Experiments 2 and 3), especially in individuals with schizotypal personality traits, in the influence of facial feedback phenomena when controlling the environment. Finally, we designed our experiments to determine whether there are different reactions or tendencies among individuals.

## Materials and Methods

### Pencil in Experiments 1 and 3

We followed the procedure of Strack et al. (1988) in which a pencil was used to induce the appropriate facial responses. Subjects were instructed to hold a pencil with their teeth only or with their lips only. In order to facilitate a smiling face, participants were told to hold a pencil with the teeth only; this would mainly contract the zygomaticus major or the risorius muscles, which are part of the smiling response (Ekman and Friesen, 1982). In order to inhibit the smiling face and facilitate a pouting face, participants were told to hold a pencil with the lips only, which would contract the orbicularis oris muscle, making it impossible to contract the zygomaticus major or the risorius muscles that are used in smiling.

### Teardrop Glasses in Experiments 2 and 3

**Figure 1:**
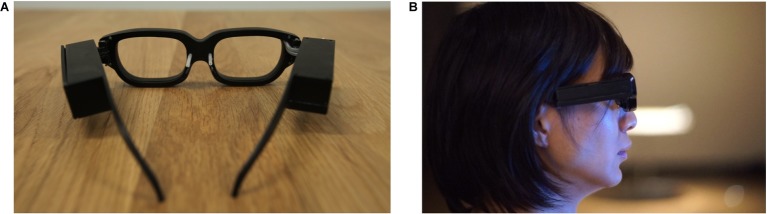
Teardrop glasses, by Yoshida (2015), http://www.shigeodayo.com/tear_drop_glasses.html.

The frames of the teardrop glasses used in the experiments were created *via* a 3D printer (see Figure 1). They weighed about 72 g and were 175 mm × 150 mm × 40 mm in size. Two adjustable pipes were installed beside each spectacle frame to allow dispensation of the tear-like liquid. Since tears drop from the corners of the eyes and often run along the nose, we ensured that the tear-like liquid was dispensed from the inner side of the frames to better emulate the experience of actual crying. The teardrop glasses are installed with a special module that utilizes an infrared communicator and changes in the brightness of the module signals when the tear-like liquid should be dispensed. More specifically, when the module turns black, no liquid is dispensed, but when it turns white, the liquid is immediately dispensed. To avoid distracting participants, we set the module to change color every 2.5 s automatically in our experiments. In order to induce a negative emotion, the switch of the teardrop glasses was turned on while the switch of the teardrop glasses was turned off as a control.

### Measures

Participants rated the intensity of two general kinds of emotions, namely positive and negative emotions. More specifically, considering cultural differences in facial cognition between Asian and western populations (Ekman et al., 1987), four basic emotions (Ekman, 1999) – happy, excited, sad, and angry – were adopted. The feelings of happy and excited were selected to represent positive emotions, since they are commonly evoked by a smiling facial expression (Freitas-Magalhães, 2007). Sad and angry were selected to represent negative emotions, as they commonly related to the behavior of crying, especially in a negative sense (Kottler, 1996; Scheirs and Sijtsma, 2001). The emotional scales of the recalled positive emotional (i.e., happy or excited) and negative emotional (i.e., sad or angry) memories were rated using a five-point Likert scale (Likert, 1932) (e.g., for happiness: not happy at all; not happy; neutral; happy; and very happy).

The Schizotypy Traits Questionnaire (STA; Claridge and Broks, 1984; Gregory et al., 2003) was adopted to measure participants’ schizotypal traits. The STA is a questionnaire comprised of 37 true-false, self-report items, based on the DSM-III diagnostic criteria for schizotypal personality disorder. It has a particular focus on perceptual aberrations, which are analogous to the positive symptoms of schizophrenia (e.g., auditory hallucinations, thought insertion, and delusions of control).

### Recruitment, Ethics Approval, and Informed Consent

Participants were all recruited at the Tokorozawa campus of Waseda University, where all the experiments were conducted. Participants were chosen from volunteers who saw a poster advertising the experiment or who attended a class of one of the researchers, under the conditions that they had no history of mental illness, were mentally and physically healthy, and that their native language was Japanese.

All participants were paid for their participation. The Graduate School of Human Sciences Committee of Waseda University approved the protocol. Informed consent was obtained from all participants, who stated that the experiment was conducted by their own free will, all data regarding the behavioral experiments and results were kept separate from their personal information, their privacy was protected, and they could cease participation and withdraw their data any time they felt uncomfortable, during or after the research. After the experiment, they were debriefed, asked whether they had any questions, provided a copy of the consent form, and given a final opportunity to withdraw their data.

According to the *Ethical Guidelines for Medical and Health Research Involving Human Subjects* of Waseda University, we believe that ethical approval was not required for the study, and written informed consent was obtained from all participants.

## Experiment 1

### Participants

Forty undergraduate students from Waseda University (16 males; 24 females; mean age = 19.85 years; SD = 0.82; range: 19–21 years) were randomly allocated into two conditions: 20 were placed in a “smile” condition and 20 in a “pout” condition. Participants were tested individually.

### Procedure

In line with the experiment conducted by Strack et al. (1988), participants were randomly divided into two conditions according to the way they held the pencil. Participants in the smile condition were directed to grip the pencil with their teeth to create a smile-like facial expression, while participants in the pout condition were directed to hold the pencil with their lips without letting their teeth touch it, thus creating a pout face-like facial expression.

Before the experiment, all participants were told they would take part in an experiment intended to study facial muscles so they would not know the true purpose of the study. The instructions were as follows:

This is a study for psychomotoric coordination. Sometimes, physically impaired people use their mouths to write words, draw pictures, or perform basic tasks, which normally people would not do with their mouths. Knowing the potential to do several tasks with their mouths is important for their future lives. This experiment is just a part of the larger project, and our interest in the experiment is how tired people get when they keep opening their mouths. We would like you not to pay attention to your mouth but rather focus on the task we are going to give you.

Assistants used photographs (a man who is gripping a pencil with his teeth, or a man who is holding a pencil with his lips) to indicate to the participant how to grip the pencil properly. When participants under both conditions were holding the pencils, they were asked to recall an impressive experience that occurred at some point in their lives in 3 min without doing anything, and they were expected to recall this memory as vividly as possible, especially focusing on the emotions they were experiencing while holding the pencil. The instructions were as follows:

Here is your task. From now, you will have 3 min. During the 3 min, we would like you to recall your autobiographical memory—that is, any episodes recollected from your life. When an individual episode comes up in your mind, please think about more details, what happened, and how you felt at that time.

Subsequently, participants were asked to write that experience down on paper including as detailed as possible a description of what had happened and, more importantly, how they felt at that time. Upon finishing it, the intensities of four emotions, “happy,” “excited,” “sad,” and “angry” on their memory were rated *via* the Likert scale questionnaire.

The emotional valence (e.g., happy or sad) of the participant’s written memory was carefully compared with their reported emotional state score (e.g., happy or sad), to ensure they were reasonably consistent with each other. For memories that were not clearly described, their reported emotion states scores were selected to represent their current emotional states. By doing so, we were able to analyze the emotional valence of their emotional states while holding a pencil in the lab.

### Results

**Figure 2:**
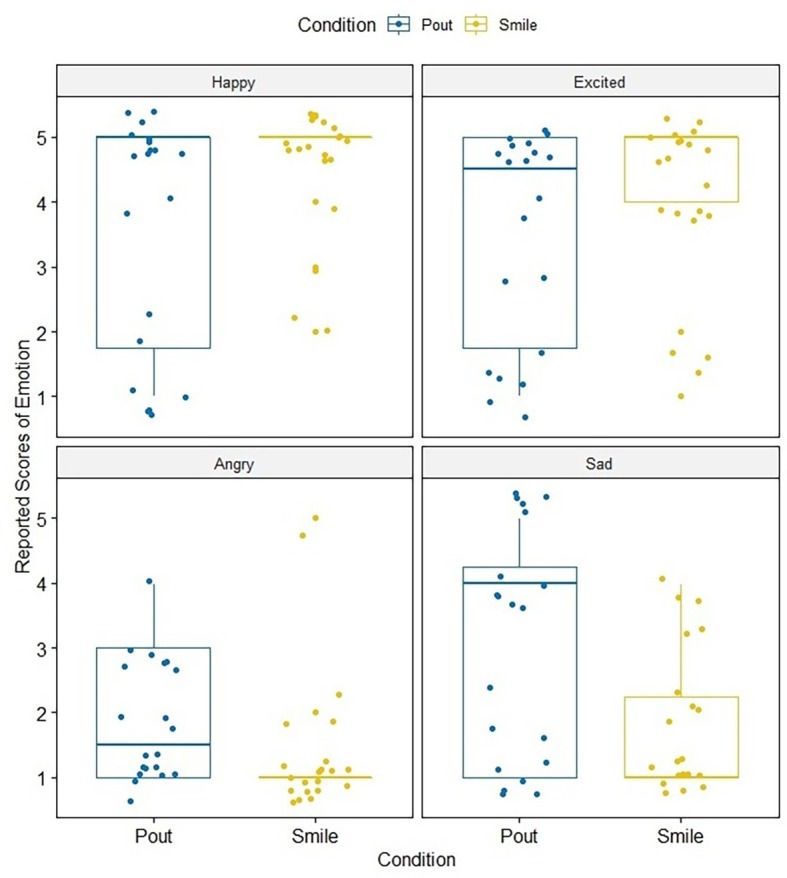
Self-reported score of emotional states (happy, excited, angry, and sad) in the smile and pout conditions of Experiment 1.

Analyses were conducted with ANOVA 4.0 online (Kiriki, 2002). Figure 2 shows the self-reported scores of happy, excited, angry, and sad emotional states of participants for both the smile and pout conditions. A mixed analysis of variance (ANOVA) with the environmental stimuli condition factor (i.e., smile vs. pout) as a between-subjects variable and the self-reported scores of emotional states of the participants (i.e., happy, excited, angry, and sad) as the within-subjects variable was conducted. There was a significant interaction [*F*(3, 114) = 5.14, MSE = 1.99, *η*^2^ = 0.14, *p* = 0.0023] between the environmental stimuli condition factor (i.e., smile vs. pout) [*F*(1, 38) = 0.04, MSE = 0.056, *η*^2^ = 0.00, *p* = 0.8392] and the self-reported scores of emotional states of the participants (i.e., happy, excited, angry, and sad) [*F*(3, 114) = 27.60, MSE = 54.86, *η*^2^ = 0.73, *p* < 0.001]. Analysis of main effect showed that the scores of happy emotional states in the smile condition (*M* = 4.55, SD = 0.97) were significantly higher than in the pout condition (*M* = 3.60, SD = 1.74) [*F*(1,152) = 4.9, *p* = 0.0277]. The scores for excited emotional states in the smile condition (*M* = 4.20, SD = 1.17) were not significantly different from those in the pout condition (*M* = 3.55, SD = 1.69) [*F*(1,152) = 2.31, *p* = 0.1304]. Negative emotions, such as the scores of angry emotional states in the smile condition (*M* = 1.85, SD = 1.10), were not significantly different from those in the pout condition (*M* = 1.90, SD = 1.00) [*F*(1,152) = 1.66, *p* = 0.2002], while the scores of sad emotional states in the smile condition (*M* = 1.85, SD = 1.11) were also significantly lower than in the pout condition (*M* = 3.05, SD = 1.63) [*F*(1,152) = 7.88, *p* = 0.0057]. Additionally, positive emotional states, such as happy and excited, were reported higher in the smile condition reported with more positive emotional autobiographical memories, while negative emotions, such as sad and angry, were reported lower, and less negative emotional-related memories were wrote. These results suggest that people who simulated a smiling face not only felt more positive emotions but also tended to recall positive memories, specifically memories that evoked positive emotions; the same pattern was not found for negative memories.

### Discussion

The result of Experiment 1 fairly proved that participants were affected by the facial expressions created by the pencil, consistent with facial feedback theory. Participants reported experiencing emotion consistent with the facial expression created by gripping the pencil with their teeth, especially in the smile condition. Moreover, they reported a congruent emotion (e.g., happy and excited) while recalling the autobiographical memory with their self-reported emotion when the embodied cognition experience occurred (e.g., a smiling facial expression). This may be caused by mood congruency effects.

Previous studies showed that emotional memories tend to be clear and detailed, regardless of whether they focus on good or bad events (Bradley et al., 1992; Christianson, 1992; Hamann, 2001; Buchanan and Adolphs, 2002). Mood congruency effects suggest that emotional memories are also better recalled when the participant is in a similar mood to that of the recalled memory (Bower, 1981). This is a type of memory bias called emotion-congruent retrieval, which specifically refers to how people remember past events or the features of past events, not as they happened but with the same emotional tone as their current state (Bower, 1981; Kensinger, 2004). Riskind (1983) similarly suggested that people tend to recall experiences where they were experiencing emotions comparable to their current mood state. The simulation view of autobiographical memory is that modality-specific states of perception, action, and introspection activated during the original experience of an event are reactivated when the experience is later represented (Niedenthal et al., 2005, 2014). This view was supported by a study where participants retrieved autobiographical memories in body-congruent and body-incongruent positions, relative to that of the original experience (Dijkstra et al., 2007). Participants were not only faster at retrieving the memory in a body-congruent position, but also retained the memory better (Dijkstra and Post, 2015). In a study by Drače et al. (2015), participants were asked to recall one personal memory after being subjected to negative and neutral mood inductions. Results showed that after being exposed to the same semantic material, the recalled memories of participants in the strong negative mood condition were more negative than those in the moderate negative mood condition.

As discussed previously, negative facial expressions seemingly work differently to positive facial expressions (Alam et al., 2008). The difference in self-reported scores of positive and negative emotional states in the pout condition was not as distinct as in the smile condition. We postulate that this was related to culturally specific negative emotional expression styles, since Japanese people tend to inhibit their negative feelings to adapt to the social expectation of harmony (Matsumoto, 1991; Winton et al., 1995; Jack et al., 2012). Another explanation might be found in individual differences in regard of negative emotion and its expression. In the next phase, the self-reported scores of positive and negative emotional states when using the teardrop glasses were compared to examine if the situation would be different in an indirective emotion-evoked circumstance and, more importantly, to investigate whether individuals with schizotypal traits would be more strongly affected in indirective environmental simulated conditions, such as teardrop glasses.

## Experiment 2

### Participants

Seventy undergraduate students from Waseda University (37 males; 33 females; mean age = 19.76 years; SD = 0.86; range: 19–21 years) participated in this experiment. First, they were randomly allocated into two conditions: the “crying” condition (35 participants) and “no-crying” condition (35 participants). Participants were tested individually. All participants answered the STA questionnaire after the experiment.

### Procedure

All participants were equipped with teardrop glasses during the experiment, after which they were allocated to the crying and no-crying conditions. In the former condition, participants wore teardrop glasses that dispensed liquid every 2.5 s, while participants in the latter condition wore teardrop glasses that did not dispense liquid.

Participants were told how the teardrop glasses worked. To prevent participants from becoming suspicious of the study purpose, they were given the following explanations. In the crying condition, participants were told, “These are a pair of glasses frames in development. Liquid is dispensed from the glasses. People who wear these teardrop glasses will look like they are crying from the viewpoint of others who are looking at them. In the future, the glasses will be used to study empathy—specifically, how people feel when they see others crying. This experiment is just a pilot study to know how comfortable the glasses are while people wear them and focus on thinking about another thing.” In the no-crying condition, participants were told, “These are a pair of glasses frames in development. Liquid is dispensed from the glasses. People who wear these teardrop glasses will look like they are crying from the viewpoint of others who are looking at them. In the future, the glasses will be used to study empathy—specifically, how people feel when they see others crying. This experiment is just a pilot study to know whether the frame is light enough to feel comfortable when people wear them. That is, liquid will not come out in this experiment. We want to know how comfortable they are while people wear them and focus on thinking about another thing.”

While wearing the glasses, participants in both conditions were asked to perform the recall task as in Experiment 1 (recalling their autobiographical memory). Subsequently, participants were asked to write that experience down on paper including as detailed as possible a description of what had happened and how they felt at that time. Upon finishing it, the intensities of four emotions, “happy,” “excited,” “sad,” and “angry” on their emotional states were rated *via* the Likert scale questionnaire. After filling out the STA, they were dismissed.

The emotion states of the written-down memory and reported content were carefully compared, as in study 1.

### Results

**Figure 3:**
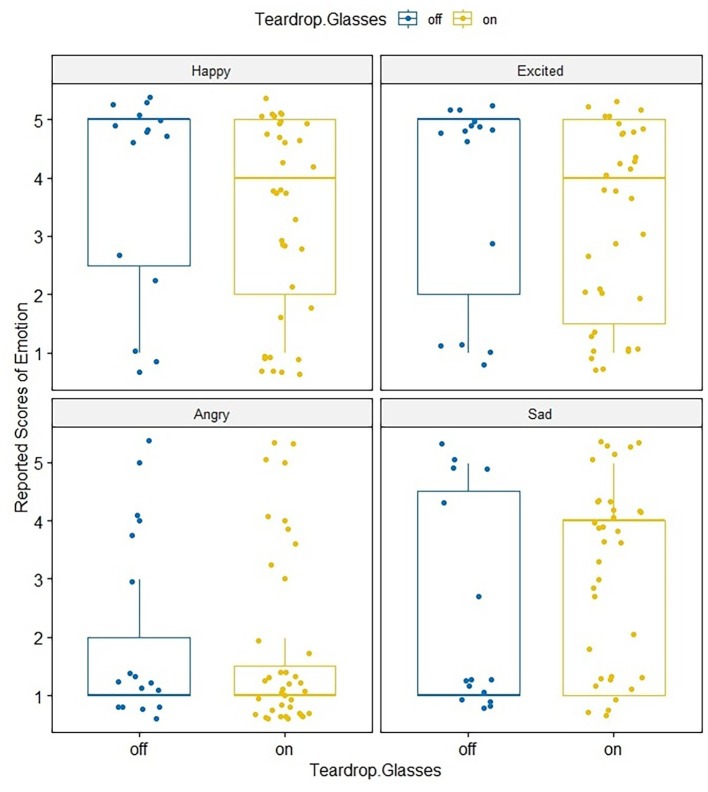
Self-reported score of emotional states (happy, excited, angry, and sad) in the crying and no-crying conditions of Experiment 2.

The self-reported scores of emotional states, rather than the written-down autobiographical memories, were analyzed to avoid ambiguous emotional descriptions. Analyses were conducted with ANOVA 4.0 online (Kiriki, 2002). Positive crying (e.g., tearing up when feeling happy) was not reported in this experiment. Figure 3 shows the self-reported scores of happy, excited, angry, and sad emotional states of participants for in the crying and no-crying conditions. Again, a mixed ANOVA was conducted with the condition (crying vs. no-crying) as a between-subjects variable and the self-reported scores of emotional states (happy, excited, angry, and sad) as a within-subjects variable. There was no significant main effect for the crying and no crying conditions [*F*(1, 68) = 0.67, *η*^2^ = 0.00, *p* = 0.42] or the self-reported scores of emotional states (happy, excited, angry, and sad) [*F*(3, 68) = 12.39, *η*^2^ = 0.18, *p* = 0.42], nor was there significant interaction [*F*(3, 204) = 0.72, *η*^2^ = 0.01, *p* = 0.0.54]. The scores for the four emotional states, happy (crying condition: *M* = 3.63, SD = 1.76; no-crying condition: *M* = 3.37, SD = 1.59), excited (crying condition: *M* = 3.57, SD = 1.83; no-crying condition: *M* = 3.23, SD = 1.61), angry (crying condition: *M* = 1.94, SD = 1.45; no-crying condition: *M* = 1.71, SD = 1.34), and sad (crying condition: *M* = 2.63, SD = 1.84; no-crying condition: *M* = 3.09, SD = 1.50), were not significantly different between the crying and no-crying conditions. This means that when comparing the crying and no-crying conditions, people whose teardrop glasses dispensed tears did not experience stronger negative emotions (i.e., angry and sad), than did people whose glasses did not dispense tears.

**Figure 4:**
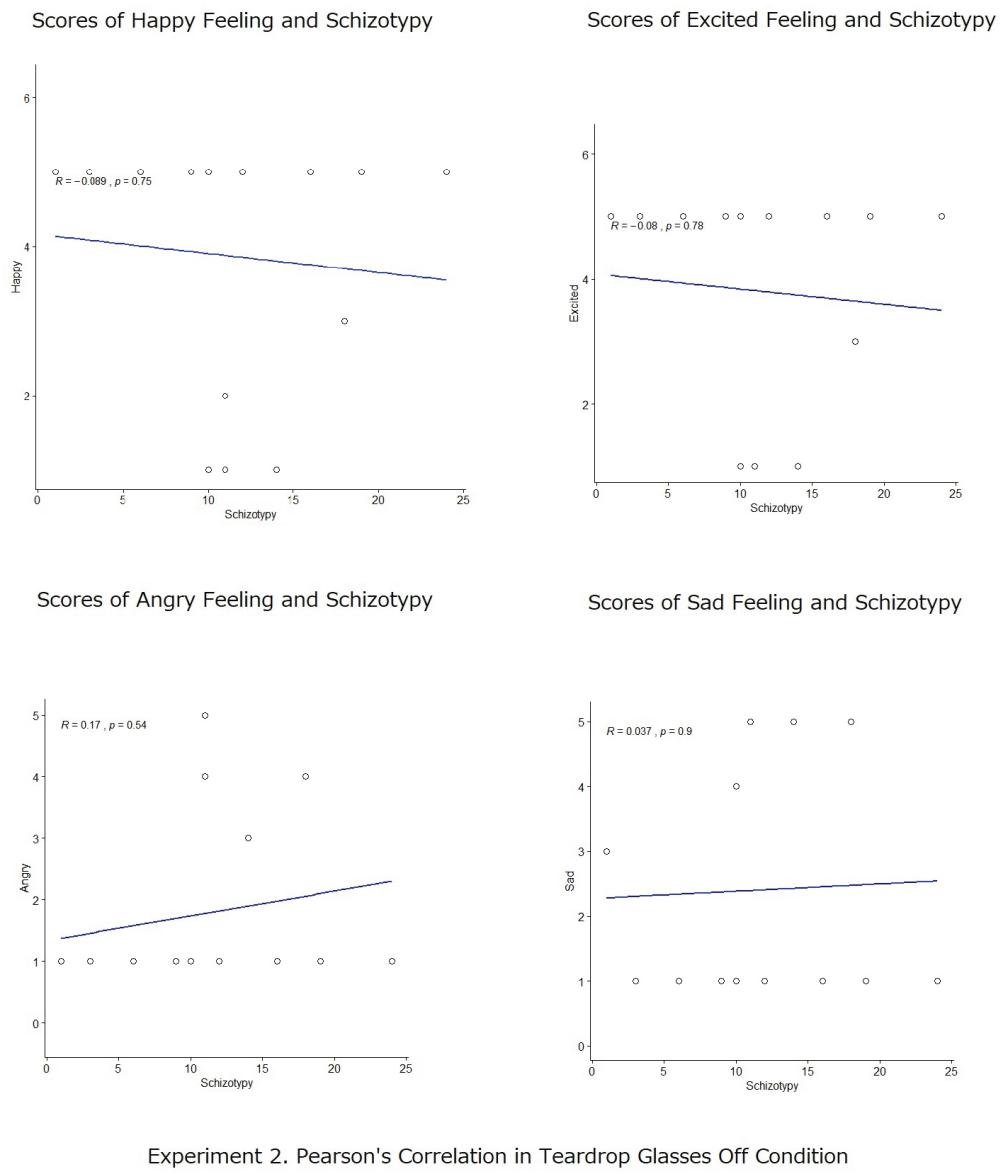
Pearson’s correlation analyses between participants’ scores in schizotypy (STA) and the self-reported scores of emotional states (happy, excited, angry, and sad) in the no-crying conditions.

**Figure 5:**
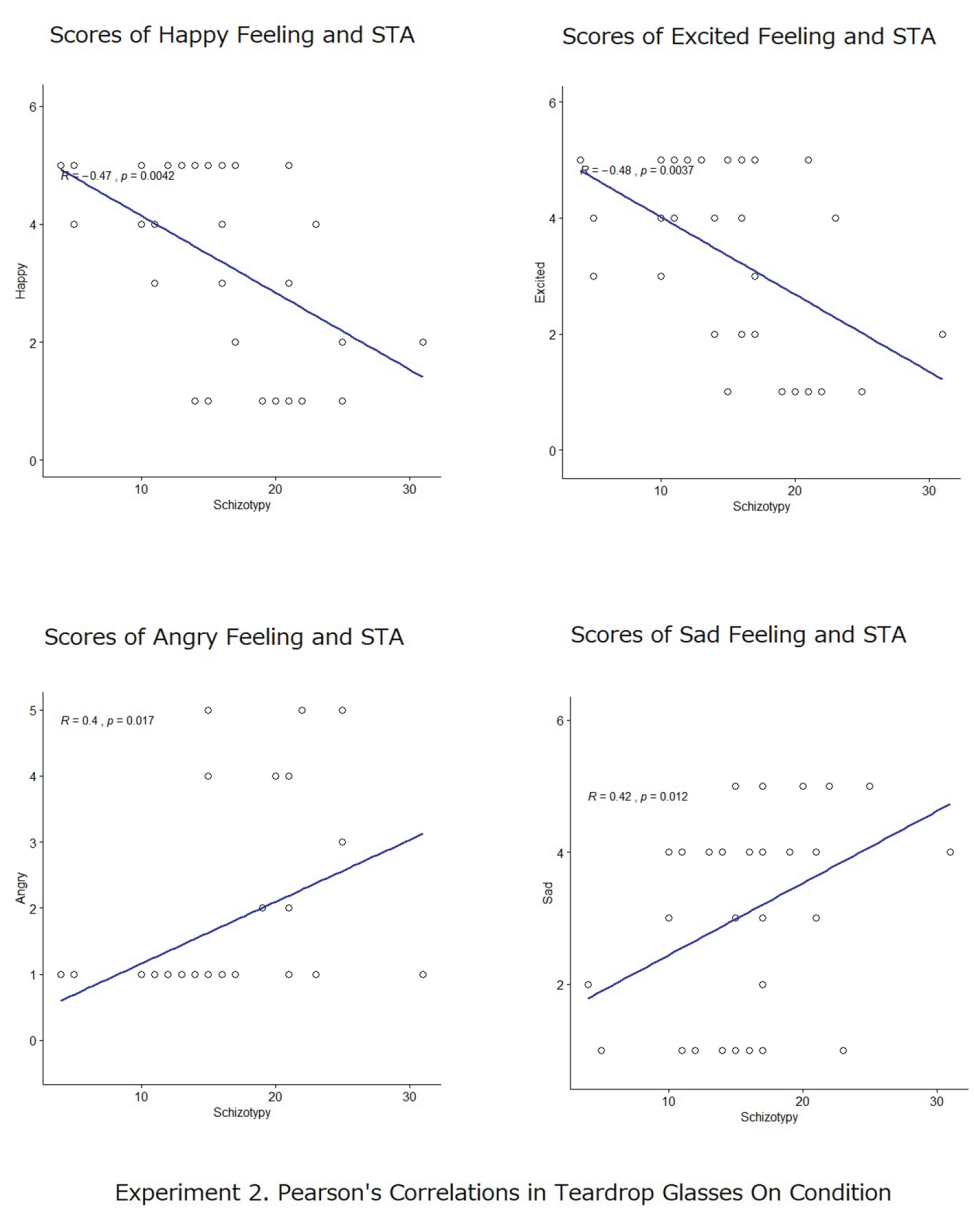
Pearson’s correlation analyses between participants’ scores in schizotypy (STA) and the self-reported scores of emotional states (happy, excited, angry, and sad) in the crying conditions.

Subsequently, Pearson’s correlation analyses were also conducted to measure the degree of the relationship between participants’ scores in schizotypy (STA) and the self-reported scores of emotional states in both the crying and the no-crying conditions (Figures 4, 5). In the no-crying condition, the Pearson’s correlations between STA score and the rates of positive emotional scales and between STA score and the rates of negative emotional scales for recalled action statements were not significant (happy: *r* = −0.06, *p* = n.s.; excited: *r* = 0.05, *p* = n.s.; angry: *r* = 0.17, *p* = n.s.; sad: *r* = 0.05, *p* = n.s.); meanwhile, those in the crying condition were significant (happy: *r* = −0.47, *p* < 0.001; excited: *r* = −0.48, *p* < 0.001; angry: *r* = 0.40, *p* < 0.01; sad: *r* = 0.42, *p* < 0.42). This analysis shows that in crying condition, participants with high scores in STA reported more negative emotion (i.e., angry and sad) than positive emotion (i.e., happy and excited).

Additionally, a percentage bend correlation (Mair and Wilcox, 2019) was also conducted with the free software R 3.6.1 to double check the analysis. The result was similar to that of the Pearson’s correlation. Percentage bend correlations between the STA score and the rates of positive emotional scales and between the STA score and the rates of negative emotional scales for recalled action statements were not significant (happy: *r* = −0.1214, *p* = 0.6663; excited: *r* = −0.112, *p* = 0.6910; angry: *r* = 0.2776, *p* = 0.3164; sad: *r* = 0.0996, *p* = 0.7241); however, those in the crying condition were significant (happy: *r* = −0.4809, *p* = 0.0035; excited: *r* = −0.501, *p* = 0.0022; angry: *r* = 0.4611, *p* = 0.0053; sad: *r* = 0.4173, *p* = 0.0126).

### Discussion

These results show that the higher the STA score, the more participants tended to report negative emotions, such angry and sad, to recall more negative events under the condition where tear-like liquid was dispensed from the glasses. The results suggest that participants high in schizotypy were more influenced by the teardrop glasses than those low in schizotypy, suggesting that individuals’ personality traits affected the result. The sense of ownership/agency, which is “the subjective awareness of initiating, executing, and controlling one’s own volitional actions in the world,” also refers to the ability to recognize oneself as the agent of a behavior, and the way the self establishes itself as an entity independent from the external world (Jeannerod, 2002),” was considered to be related to the results. There is some evidence for a similar relation between schizotypy and an individual’s sense of agency/ownership. Kallai et al. (2015) found that people with high schizotypy, including feelings of depersonalization when the rubber hand illusion was induced, tended to have higher interpersonal sensitivity and vulnerability scores. Asai et al. (2011) examined the relationship between individual differences in the rubber hand illusion and empathic and schizotypal personality traits, as the existing literature suggested that schizophrenic patients would be more susceptible to the illusion. The results showed that people who experience a stronger rubber hand illusion may have both stronger empathic and schizotypal personality traits. This finding might also be related to empathic functioning, which is what allows us to simulate behavior observed in others.

In the next phase, an experiment within participants was conducted, expected to provide more support for why some participants were not affected by the teardrop glasses and whether or not schizotypal personality traits could provide a satisfactory explanation.

## Experiment 3

### Participants

Sixty-one undergraduate students from Waseda University (30 males; 31 females; mean age = 19.49 years; SD = 1.49; range: 18–21 years) participated in this experiment. Thirty-one of them were allocated randomly to a “smile-crying” condition and the other 30 to a “crying-smile” condition. Participants were tested individually. All participants answered the STA questionnaire after participating in the experiment.

### Procedure

All participants were asked to visit the experimental setting twice, separated by a one-week interval (Day 1 and Day 2). On Day 1, participants in the smile-crying condition were told to hold a pencil between their teeth to create a smile-like facial expression and recall their autobiographical memory under the same instructions with those in the smile condition in Experiment 1. After a week, they returned to continue the experiment on Day 2. At this session, they completed the entire procedure of the crying condition in Experiment 2. Participants in the crying-smile condition completed the same procedures but in reverse order (i.e., the crying condition first on Day 1, followed by the smiling condition on Day 2). They completed the four emotional scales (i.e., happy, excited, angry, and sad) *via* a Likert scale at the end of each task.

### Results

**Figure 6:**
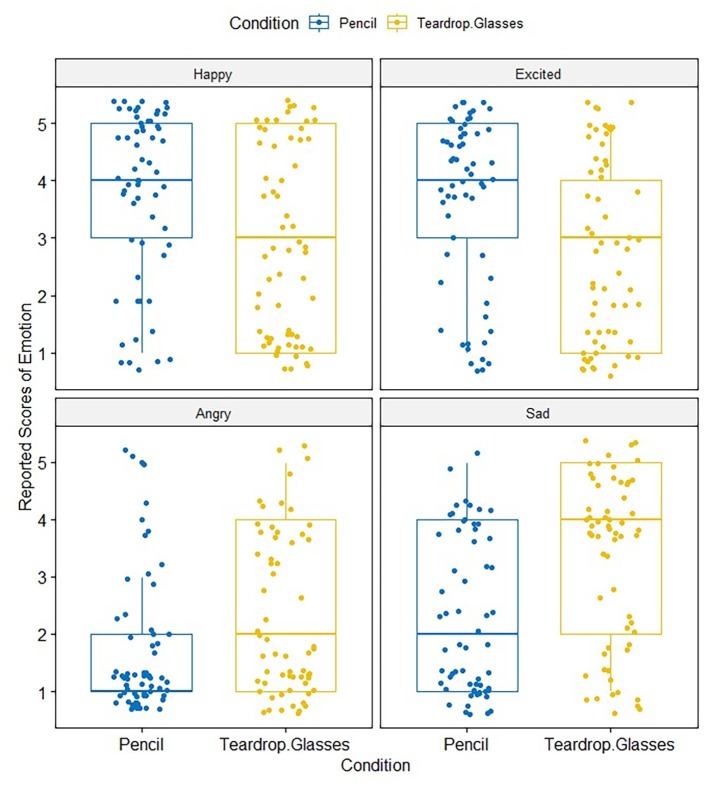
Self-reported score of emotional states (happy, excited, angry, and sad) in the smile (pencil) and crying (teardrop glasses) conditions of Experiment 3.

Figure 6 shows the self-reported scores of four types of emotional states – happy, excited, angry, and sad – in the smiling and crying conditions. A two-way ANOVA, conducted with ANOVA 4.0 online, was conducted with condition (smile vs. crying) and the scores of emotional states (happy, excited, angry, and sad) as within-subjects’ factors. We observed a significant interaction [*F*(3, 180) = 18.86, MSE = 32.23, *η*^2^ = 0.31, *p* < 0.001] between the smile and crying conditions [*F*(1,60) = 0.02, MSE = 0.008, *η*^2^ = 0.00, *p* = 0.9029] and the self-reported emotional state (i.e., happy, excited, angry, and sad) [*F*(3, 180) = 13.31, MSE = 45.69, *η*^2^ = 0.45, *p* = <0.001]. Analysis of main effects showed that the scores of happy emotional states in the smile condition (*M* = 3.72, SD = 1.45) were significantly higher than in the crying condition (*M* = 2.89, SD = 1.66) [*F*(1, 240) = 15.03, *p* = 0.0001]. The scores for excited emotional states in smile condition (*M* = 3.66, SD = 1.49) were also significantly higher than in the crying condition (*M* = 2.75, SD = 1.55) [*F*(1,240) = 17.49, *p* < 0.001]. Negative emotions, such as angry emotional states, in the smile condition (*M* = 1.62, SD = 1.12) were significantly lower than in the crying condition (*M* = 2.30, SD = 1.38) [*F*(1, 240) = 9.72, *p* = 0.0020]. The scores for sad emotional states in the smile condition (*M* = 3.65, SD = 1.50) were also significantly lower than in the crying condition (*M* = 2.75, SD = 1.55) [*F*(1, 240) = 25.95, *p* < 0.001]. Additionally, the order of the smile and crying conditions was analyzed; we found no significant interaction related to the order [*F*(1, 59) = 1.95, MSE = 1.91, *p* = 0.1677].

**Figure 7:**
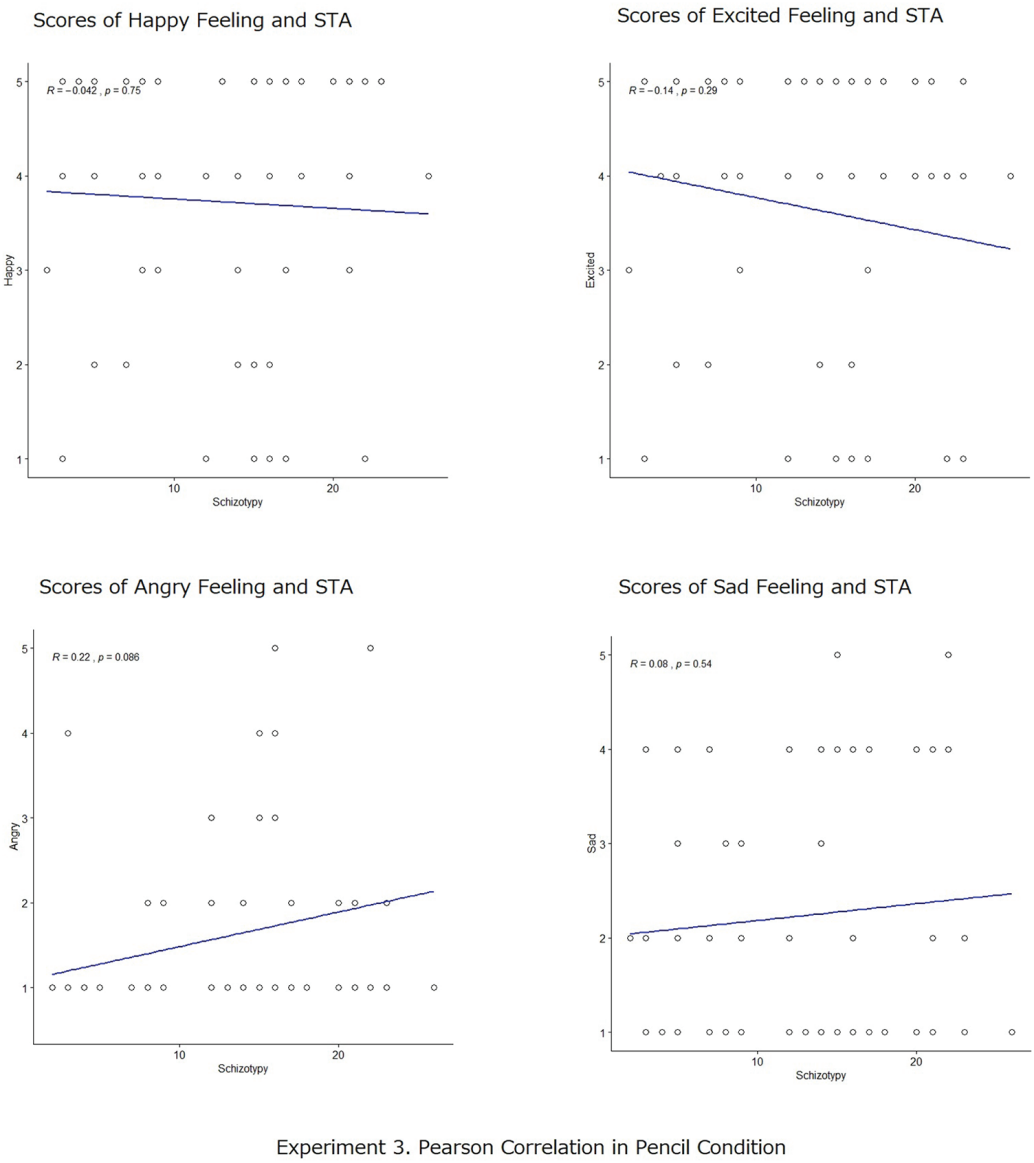
Pearson’s correlation analyses between participants’ scores in schizotypy (STA) and the self-reported scores of emotional states (happy, excited, angry, and sad) in the smile conditions.

**Figure 8:**
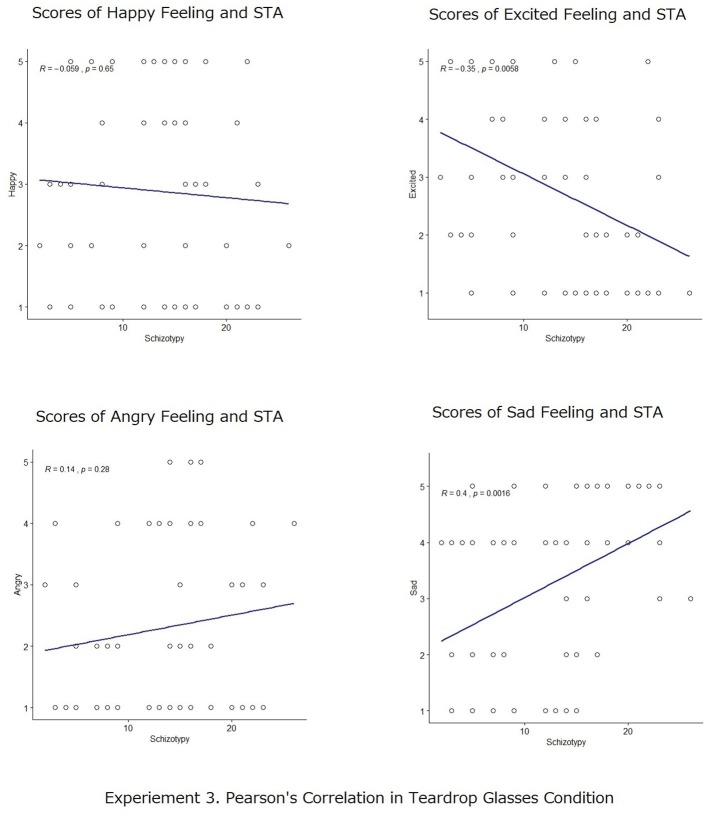
Pearson’s correlation analyses between participants’ scores in schizotypy (STA) and the self-reported scores of emotional states (happy, excited, angry, and sad) in the crying conditions.

Pearson’s correlation analyses were also conducted to measure the degree of the relationship between participants’ scores in schizotypy (STA) and the rates of positive and negative emotional scales for recalled action statements both in the smiling and in the crying conditions. The results are shown in Figures 7, 8. In the smiling condition, there was no significant correlation between STA scores and the scores of positive emotional states (happy: *r* = −0.004, *p* = n.s.; excited: *r* = 0.14, *p* = n.s.); nor between STA scores and the scores of negative emotional states (angry: *r* = 0.22, *p* = n.s.; sad: *r* = 0.08, *p* = n.s.). Meanwhile, in the crying condition, there were significant correlations according to the Pearson’s correlation coefficient between STA scores and the scores of the excited emotional states (*r* = −0.35, *p* < 0.001), indicating strong negative correlations between the variables as well as the scores of sad emotion states (*r* = 0.40, *p* < 0.001), indicating strong positive correlations between the variables. While no significant correlations were found in this analysis for STA scores and the scores of happy emotional states (*r* = −0.06, *p* = n.s.) and STA scores and the scores of angry emotional states (*r* = 0.14, *p* = n.s.).

Again, percentage bend correlation (Mair and Wilcox, 2019) was conducted to check the results. It was found that in the crying condition, there were significant correlations between STA scores and the scores of the excited emotional states (*r* = −0.3501, *p* = 0.0057), indicating strong negative correlations between the variables, as well as the scores of sad emotion states (*r* = 0.4161, *p* = 0.0009), indicating strong positive correlations between the variables. No significant correlations were found in this analysis for STA scores and the scores of happy emotional states (*r* = −0.0678, *p* = 0.6035) and STA scores and the scores of angry emotional states (*r* = 0.1302, *p* = 0.3172).

### Discussion

These findings successfully replicated Experiments 1 and 2 within participants, further suggesting that participants high in schizotypy were more affected by wearing the teardrop glasses – that is, they tended to recall memories related to that negative emotion – while there was no relationship between the emotional scales and schizotypy when holding a pencil between their teeth. Moreover, participants with low schizotypy, indicated through low scores on the STA questionnaire, showed a different trend in the smile and crying conditions than those with high schizotypy. In the crying condition, they scored higher in the negative emotional state of the recalled autobiographical memory, which might suggest that they remembered more negative emotion-related memories consistent with the “teardrop” condition.

## General Discussion

“Emotions are complex perceptions created in the mind of a perceiver when people make meaning of basic visceral feelings in a given context (James, 1884).” James argued that people instead experienced various elemental biological and psychological states, from which they constructed a personal emotional experience for themselves (Lindquist, 2013). Meanwhile, emotion theorists have proposed that producing facial expressions and receiving sensory feedback from the face modulates the intensity of—or, in the strong formulation of the hypothesis, creates—emotional experience. According to the facial feedback hypothesis, congruent facial expressions enhance corresponding feelings, whereas the inhibition of these expressions or the display of expressions incongruent with the felt emotion attenuates those feelings (Niedenthal, 2007). Several experiments have found support for this hypothesis; for example, inhibition of facial expressions attenuated self-reported pleasantness ratings, whereas amplification of these expressions increased pleasantness ratings (Lewis and Bowler, 2009; Hyniewska and Sato, 2015). Furthermore, people instructed to imitate angry facial expressions had greater pupillary dilation and skin conductance than did participants who were simply viewing angry expressions (Lee et al., 2013). We are interested in how stronger facial feedback would affect people’s mood, or whether it would only affect their current emotion. We noticed that there were few studies exploring how facial feedback theory works in directive and indirective stimuli, thus we decided to further investigate the facial feedback effect and different environmental stimuli. Our study’s findings confirmed that facial feedback effect occurs more strongly under some conditions and emotional experience influences facial feedback more strongly than do other types of experiences, similar to what Coles et al. (2019) found in their research.

Considering that the paradigms we adopted in these studies are controversial, as we mentioned in the “Introduction” section, we designed our experiments based on the facial feedback hypothesis in a carefully controlled situation. We asked participants to imagine rather than watch subjects, and we did not monitor them with a camera. We also focused on the different emotional experiences caused by personality traits in the phenomenon of facial feedback and how they relate to the participants’ autobiographical memories. We also employed a newly created tool, teardrop glasses to simulate negative emotion, as well as a traditional method of using a pencil to stimulate positive (smile) and negative (pout) emotions, in the investigation of embodied cognition and environmental stimuli.

We found that using a directed approach (gripping a pencil with teeth/lips) while recalling emotional biographical memory could possibly evoke participants’ positive (e.g., happy and excited)/negative (e.g., angry and sad) emotions associated with that emotion. We believe that it also provides evidence for facial feedback theory and implies that the congruency hypothesis (Riskind, 1983) not only applies to intentionally stimulated emotions but also to emotions evoked by external forces. The results are consistent with a similar study conducted by Baumeister et al. (2015), who concluded that a mask blocking facial expressions influenced participants’ performance during both encoding and retrieval of emotional items from memory. Embodied cognition has been linked to specific behavior such as facial mimicry and eye gaze (Niedenthal et al., 2010).

The evidence concerning the teardrop glasses is more complicated. Compared to participants gripping a pencil (“the smiling face is my own smile”), participants wearing teardrop glasses do not physically interact with the tool (no facial muscle was adopted), which means you have to have a sense of ownership for the tears on your cheeks (“the tears on my cheeks are our own tears”). Therefore, it is harder to have a sense of ownership over the “tears.” Linked to the result of Experiment 1, where no significant differences in positive and negative rates were found in the pout condition, a possible explanation could be that the lower effect depended on the valence of the induced emotion in relation to its congruence. The results of our experiment investigating the effect of the teardrop glasses on memory (Experiments 2 and 3) were robust, showing that while participants who scored low in schizotypy reported little effect from wearing the teardrop glasses, participants with high schizotypy reported a much greater effect. People with schizophrenia compared to healthy people (Mirucka, 2016), or people with high schizotypy compared to those with low schizotypy (Asai et al., 2011), are more likely to experience the rubber hand illusion. As those previous studies, results we obtained in this research can be attributed to schizophrenia’s or high-schizotypy’s disruptions in the sense of body ownership. Nishio et al. (2018) investigated the relationship between body ownership and facial feedback phenomena. In that study, when participants felt ownership of a robot, they were likely to interpret the facial expression of the robot as their own emotional situation (Nishio et al., 2018), suggesting that having ownership of your facial expression (“The smiling face is my own”) makes you reason that the facial expression comes from your emotional situation. Another explanation could be the affective traits and variate emotion recognition of schizotypy (Edwards et al., 2002). Horan and Blanchard (2003) stated that schizophrenic patients might experience stress related to negative affectivity, with similar decreases in positive mood in a controlled group during a role-play test of social encounters, requiring assertive or affiliative social skills.

These experiments conducted in this study provided evidence for facial feedback theory, revealing that the mechanism of the effect of facial feedback might be complicated. The effect could be influenced by the environmental setting, such as the presence or absence of a camera, the process of stimulating emotion, such as remembering an emotional autobiographic memory, and individual differences, such as schizotypal personality traits. A recent study exploring embodied experience in a virtual environment with a similar conclusion stated that how strongly a participant became immersed in a VR was related to their personal traits, especially the way they view and accept the given story in VR (Shin, 2018). Tschacher et al. (2017) discussed the implications of embodiment and schizophrenia, explicitly saying they believed that understanding people with schizophrenia is particularly pertinent in embodiment studies because most of the symptoms and signs of schizophrenia could be driven by false perceptions and beliefs about the cause of sensations, which is consistent with previous studies of sense of agency/ownership. We believe that this study could offer some recommendations for body psychotherapy, such as how to improve the construction of therapeutic environments (Röhricht et al., 2014), and the psychotherapy of schizophrenia patients (Gallese and Ferri, 2015).

Our study adds to the literature on the relationship between simulated emotion and emotion of recalled autobiographical memory, suggesting that external equipment, such as teardrop glasses, could affect the recall of individuals’ emotional autobiographical memory. In a previous study, patients suffering from major depressive disorder showed reduced symptom severity after receiving Botox injections to the muscles involved in eyebrow furrowing, a movement associated with negative emotions (Finzi and Rosenthal, 2016). Accordingly, our results might be applied to the clinical field to help people suffering from emotional problems or people with schizophrenia (Helt and Fein, 2016).

The results of these experiments provide novel insight into embodied cognition and its association with emotional autobiographical memory. However, the cognitive processes that arise when someone is wearing teardrop glasses (an indirect process) might not be the same as what happens when gripping a pencil in one’s teeth (a direct process). Future research should therefore employ similar processes, such as incendiary reflection (Yoshida, 2013) and teardrop glasses, to make comparisons more objective.

## Data Availability Statement

All datasets generated for this study are included in the article/supplementary material.

## Ethics Statement

Ethics approval was not required for this study as per the Waseda University’s Ethical Guidelines for Medical and Health Research Involving Human Subjects and applicable national regulations. Written informed consent was obtained from all participants.

## Author Contributions

YL conceived and designed the analysis, collected the data, performed the analysis, and wrote the manuscript. KS conceived and designed the analysis, collected the data, and performed the analysis. SY invented the teardrop glasses, made instructions how to use them, and fixed them when they didn’t work. SY also checked the section “Methods and Discussion.” ES conceived and designed the analysis, contributed to data and analysis tool, revised the first version of the manuscript, and performed the final check of the manuscript.

### Conflict of Interest

The authors declare that the research was conducted in the absence of any commercial or financial relationships that could be construed as a potential conflict of interest.
